# Shedding light on cell compartmentation in the candidate phylum Poribacteria by high resolution visualisation and transcriptional profiling

**DOI:** 10.1038/srep35860

**Published:** 2016-10-31

**Authors:** Martin T. Jahn, Sebastian M. Markert, Taewoo Ryu, Timothy Ravasi, Christian Stigloher, Ute Hentschel, Lucas Moitinho-Silva

**Affiliations:** 1 Julius-von-Sachs Institute for Biological Sciences, University of Würzburg, Würzburg, 97082, Germany; 2 Marine Microbiology, GEOMAR Helmholtz Centre for Ocean Research, Kiel, 24105, Germany; 3 Division of Electron Microscopy, Biocenter, University of Würzburg, 97074, Würzburg, Germany; 4 Division of Biological and Environmental Sciences & Engineering, King Abdullah University of Science and Technology, Thuwal, 23955-6900, Kingdom of Saudi Arabia; 5 Christian-Albrechts-University of Kiel, Germany; 6 School of Biotechnology and Biomolecular Sciences & Centre for Marine Bio-Innovation, University of New South Wales, Sydney, 2052, Australia

## Abstract

Assigning functions to uncultivated environmental microorganisms continues to be a challenging endeavour. Here, we present a new microscopy protocol for fluorescence *in situ* hybridisation-correlative light and electron microscopy (FISH-CLEM) that enabled, to our knowledge for the first time, the identification of single cells within their complex microenvironment at electron microscopy resolution. Members of the candidate phylum Poribacteria, common and uncultivated symbionts of marine sponges, were used towards this goal. Cellular 3D reconstructions revealed bipolar, spherical granules of low electron density, which likely represent carbon reserves. Poribacterial activity profiles were retrieved from prokaryotic enriched sponge metatranscriptomes using simulation-based optimised mapping. We observed high transcriptional activity for proteins related to bacterial microcompartments (BMC) and we resolved their subcellular localisation by combining FISH-CLEM with immunohistochemistry (IHC) on ultra-thin sponge tissue sections. In terms of functional relevance, we propose that the BMC-A region may be involved in 1,2-propanediol degradation. The FISH-IHC-CLEM approach was proven an effective toolkit to combine -omics approaches with functional studies and it should be widely applicable in environmental microbiology.

The majority of microorganisms in nature remains uncultivated and is commonly referred to as “microbial dark matter”^1^. This uncultivated microbial majority holds new insights into biology and biotechnology as well as evolution^2^,^3^,^4^. Cultivation-independent high throughput sequencing surveys have provided comprehensive insights towards diversity and function of the microbial dark matter. However, these insights fail to provide spatial information with respect to bacterial function in its microenvironment. While electron microscopy is an established method to study structure and ultrastructure, fluorescence microscopy allows the identification of specific molecules such as taxonomic marker genes^5^ or proteins^6^. Correlative light and electron microscopy (CLEM) combines the advantages of both modalities allowing to put molecular identity into structural context^7^. CLEM is therefore predestined to shed light on uncultivated prokaryotes thriving in complex microbiomes. Marine sponges for example contain massive amounts of microorganisms within their mesohyl matrix, which may contribute up to 35% of the animal’s biomass^8^,^9^,^10^. Members of at least 47 bacterial phyla and archaeal lineages were so far identified by high-throughput sequencing technologies within sponge hosts^11^,^12^,^13^. The candidate phylum Poribacteria is among the predominant microorganisms in these microbial consortia^14^,^15^. Much of our knowledge about their genomic potential was obtained by single-cell genome analyses^16^,^17^,^18^. This approach revealed details of their potential primary and secondary metabolism, including the description of a complex carbon degradation enzymatic repertoire^17^, as well as putative symbioses factors^16^,^18^. Since their first description, Poribacteria were suggested to display cellular compartmentalisation. Few experimental findings support this idea, including the observation of ring-shaped Poribacteria-specific FISH (fluorescence *in situ* hybridisation) signals^19^ and the presence of protein shell genes^16^. Structurally, protein shells can form bacterial compartments (BMC) and gas vesicles^20^,^21^,^22^. The ability for compartmentation is widespread in bacteria (reviewed in Kerfeld and Erbilgin^23^). Bacterial compartments provide confined biochemical environments within the cell where enzymatic reaction conditions are optimised, nutrients and volatiles are stored, and toxic compounds are isolated^24^,^25^,^26^,^27^.

In the present study, we aimed to resolve the ultrastructure as well as the transcriptional activity profile of poribacterial symbionts of marine sponges. To achieve this we standardised transcriptome retrieval from metatranscriptomes and present a novel protocol that extends the principles of array tomography^28^ by combining fluorescence *in situ* hybridisation (FISH) and immunohistochemistry (IHC) with scanning electron microscopy (SEM).

## Results

### High resolution visualisation and 3D reconstruction of Poribacteria

**Figure 1:**
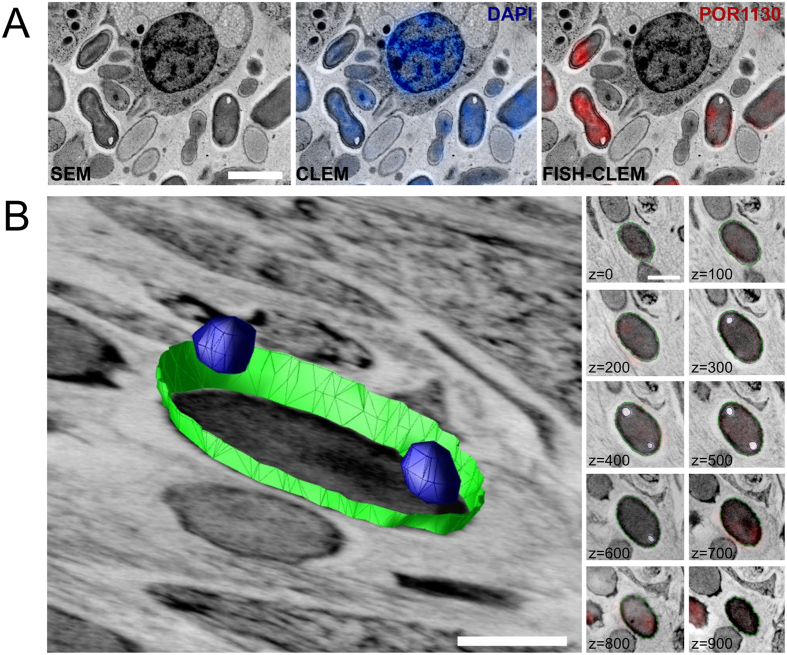
Identification of Poribacteria cells in the sponge microbiome using FISH-CLEM. (**A**) Scanning electron microscopy images (SEM) were correlated with fluorescence signal of the nucleotide stain DAPI (blue; CLEM) and the Poribacteria specific 16S rRNA probe POR1130 (red; Alexa546; FISH-CLEM) allowing the identification of microbes within the host in close proximity to a sponge cell at ultrastructure resolution. Separate channels are shown in Figure S1. (**B**) Three-dimensional reconstruction of a representative Poribacteria cell. Polar spherical structures (blue), which represent about 2% of cell volume, are typically observed at different z-intervals. Cell envelope is shown in green. Right panel displays FISH-CLEM micrographs used as basis for the reconstruction, where consistent POR1130 signals were observed across 10 consecutive slices totaling 1 μm of depth in z-dimension (z-values in nm). Scale bars, 2 μm (**A**) and 500 nm (**B**).

The FISH-CLEM method enabled the taxon-specific identification of bacterial cells at ultrastructural resolution. Poribacteria probe (POR1130, Alexa546) signals co-localised with DAPI signals and microbial cells from electron micrographs of *Aplysina aerophoba* mesohyl (Fig. 1A; Supplementary Figure S1). On the average of four areas, 21.8% (±2.9 s.d.; 792 of 2,697) of the prokaryotic cells, detected by DAPI, emitted also poribacterial probe signal. Besides, 5.4 (±1.8) poribacterial cells per sponge cell (792/154) were observed at a density of 32.8*10^3^ (±5.0 × 10^3^) cells/mm^2^. Poribacterial cells showed a consistent morphotype that appeared ovoid-shaped, with 1.5–2.2 μm in length and 0.9–1.2 μm in width. At the poles of poribacterial cells, intracellular structures of low electron density were consistently observed. Multiple structures per cell pole were observed only rarely (<1% of cells). The cellular morphology of Poribacteria was further investigated by array tomography of FISH-CLEM micrographs. The three dimensional reconstruction of seven representative poribacterial cells confirmed the presence of two bipolar intracellular structures per each cell (Fig. 1B). DAPI signals were evenly distributed within poribacterial cells without local maxima. Generally, the bipolar structures were spherical, with an average volume of 5.28*10^−3^ μm^3^ each and 168.0 nm (±25.6 nm) in diameter. Together, they made up about 1.1% (±0.7%; n = 4) of the poribacterial cell volume and did not appear to be membrane bound.

### Cell compartmentation-related genes are highly expressed

Metatranscriptomic datasets from 3 biological replicates of *Xestospongia testudinaria* were obtained and processed resulting, on average, in 43,076,693 (±7,840,577) quality filtered paired-end reads (Supplementary Table 1). These datasets were each mapped against the poribacterial single amplified genome (SAG) 3G, which was isolated from *A. aerophoba*. The retrieved Poribacteria 3G transcriptomes represented between 3.12% (1,582,793 reads; *XT*2) and 4.05% (1,417,774 reads; *XT*3) of the sequenced *X. testudinaria* metatranscriptomes. Gene expression, as estimated by FPKM values, was significantly positively correlated among biological replicates (average Pearson’s correlation coefficient P = 0.82 ± 0.11; p value < 0.001). The most abundant genes in poribacterial SAG 3G transcriptomes in *X. testudinaria* were analysed in relation to their functional classification and a set of housekeeping genes (Supplementary Data S1). We defined genes as highly expressed when expression levels were above those of housekeeping genes (average 780.1FPKM, the fold difference to this level is referred to as times FPKM_HK,_ hereafter). This included a set of 258 coding sequences (CDS) being slightly overrepresented by functionally annotated genes compared to the rest of the SAG 3G genome (76.7% vs. 68.8%).

**Figure 2:**
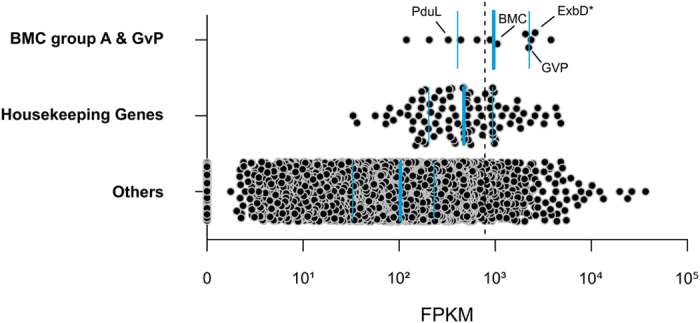
Active functions of poribacterial SAG 3G related to compartmentation in the sponge *X. testudinaria.* Expression estimations (FPKM) are shown for genes of selected functional categories. Blue horizontal lines indicate the first and third quartiles and the median (thick line) of each category. Dashed line indicates average expression level of housekeeping genes. Genes highlighted ExbD*, gas vesicle protein (GvP), BMC-shell marker, and propanediol utilization protein (PduL). The FPKM values represent the average of three biological replicates.

**Figure 3:**
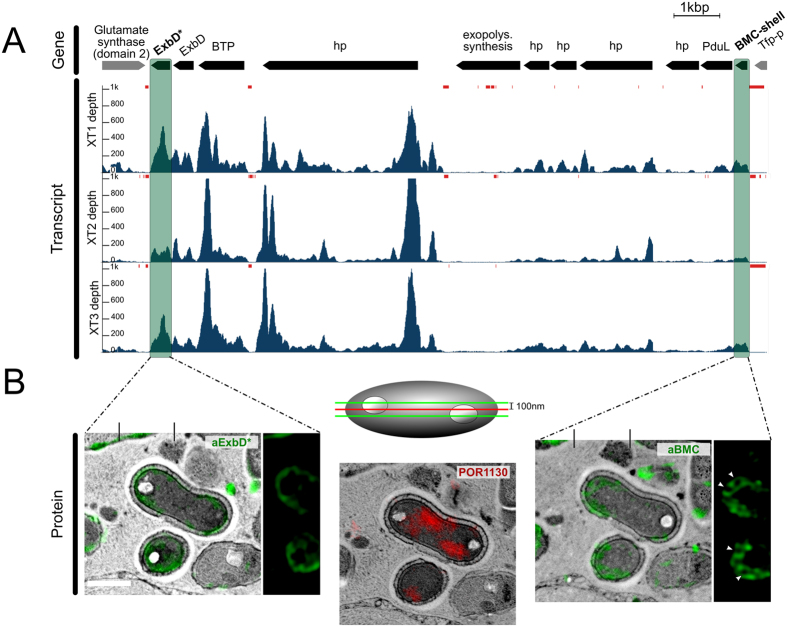
Integrative omics and microscopy study of the BMC-A genomic region. (**A**) The genomic region and transcriptional profile of poribacterial SAG 3G. Gene abbreviations are: BTP = biopolymer transport protein, hp = hypothetical protein, Tfp-p = Tfp-pilus protein. Arrows indicate gene intervals and orientation. BMC group A genes are shown in black, others in grey. The transcript coverage (reads per genomic base) for three *X. testudinaria* metatranscriptiomes (*XT*1, *XT*2, and *XT*3) was illustrated using the Integrated Genome Viewer (IGB) tool. Loci with no read coverage are highlighted by red bars. (**B**) Proteins encoded in the BMC-A, BMC-shell marker (right; green; FITC) and ExbD proteins (left; green; FITC), were localised within poribacterial cells by FISH-IHC-CLEM. Ring shaped BMC-shell signals are indicated by arrowheads. Poribacteria cells were identified at ultrastructural resolution in *Aplysina aerophoba* tissue by FISH-CLEM using the Poribacteria-specific 16S rRNA probe POR1130 (middle image, red; Alexa546 double 5′3′ labelled). Micrographs represent the same cells on 3 consecutive sections of 100 nm distance as illustrated in the scheme. Separate channels are shown in Figure S1. Scale bars, 500 nm.

Specifically, genes related to cell compartmentation involving the BMC-shell marker protein (1.3 FPKM_HK_) and gas vesicle protein (GvP) (2.9 FPKM_HK_) were found to be highly transcribed (Fig. 2). The first gene is localised in the conserved BMC-A genomic region of poribacterial SAGs^16^. Three genes coding for membrane components of biopolymer transporters found on this region were highly transcribed (Fig. 3A; Supplementary Figure S1): the ExbD* (3.3 FPKM_HK_), the ExbD (2.6 FPKM_HK_), and a protein with MotA/TolQ/ExbB proton channel family and carboxypeptidase regulatory-like domain (BTP, 4.9 FPKM_HK_). Notably, in all three sponge individuals, zero coverage was observed flanking these genes, thus indicating polycistrons, i.e. genes that are expressed in a single transcript (Fig. 3A, red bars). Additionally, the genes encoding the BMC-shell marker and the propanediol utilization protein, PduL, also appeared to be part of one polycistron.

### Subcellular localisation of cell compartmentation-related proteins

The BMC-A genomic region was further investigated by localising the proteins BMC-shell marker and ExbD* using the newly developed FISH-IHC-CLEM method. Additionally, FISH-IHC-CLEM was applied to localise the gas vesicle protein (GvP)^29^, which showed high transcription levels. Protein-specific signals were observed in the majority of the cells labelled with Poribacteria FISH probes. Specifically, BMC-specific signals were detected in 92.5% (37/40), ExbD* in 91.4% (32/35), and GvP in 100% (10/10) of Poribacteria-positive cells. The GvP protein signals were observed throughout the cytosol (Supplementary Figure S2), while the ring-shaped BMC-shell marker protein and the ExbD* protein signals were associated with cell membranes (Fig. 3B).

### Additional highly expressed functional genes in Poribacteria

Only three studies have so far reported metatranscriptome data from sponges^30^,^31^,^32^. We thus expand our analysis to provide a compilation of additional highly expressed functional genes detected here in the Poribacteria 3G transcriptomes (Supplementary Figure S3). A strong transcriptional activity was observed for genes related to: (a) central metabolism, mainly tricarboxylic acid (TCA) cycle [PATH:ko00020] (n = 2); (b) energy metabolism, including several NADH-quinone oxidoreductase subunits involved in oxidative phosphorylation [PATH:ko00190], and (c) genetic information processing, specifically genes of transcription and translation machinery (n = 43). In particular, nucleotide binding proteins such as the RNA binding domain with a RNA recognition motif (RRM; PF00076; 47.1 FPKM_HK_) and the DNA-binding protein HU (heat unstable)-beta (K03530; 25.3 FPKM_HK_) were remarkably highly transcribed. Besides, metabolism of co-factors and vitamins was abundantly represented in SAG 3G transcriptomes, in particular genes coding for pathways of folate biosynthesis ([PATH:ko00790]; folE, 1.3 FPKM_HK_, queE, 1.1 FPKM_HK_), biotin metabolism (fabF, 2.1 FPKM_HK_) and nicotinate/nicotinamide metabolism ([PATH:ko00760]; nadA, 1.4 FPKM_HK_).

Further, specific sets of genes associated with nutrient acquisition were highly transcribed, such as genes encoding enzymes that control cellular nitrogen levels, i.e., the nitrogen regulatory protein PII (17.1 FPKM_HK_) and two glutamine synthases (4.6 FPKM_HK_, 2.0 FPKM_HK_). Besides, two ammonium permeases were expressed, although at a lower level (>75^th^ FPKM percentile, 0.5 FPKM_HK_, 0.5 FPKM_HK_). With respect to sulfur metabolism, genes of the enzymatic pathway transforming thiosulfate to acetate and L-cysteine were abundant in the transcriptome, i.e. thiosulfate sulfurtransferase (TST, 1.5 FPKM_HK_), NADPH-dependent sulfite reductase (cysI, 2.0 FPKM_HK_), and cysteine synthase A (cysK, 1.2 FPKM_HK_). On the other hand, carbohydrate degradation genes, such as glycoside hydrolases^17^, were not particularly highly expressed (≤0.6 FPKM_HK_; Supplementary Data S1).

Thirdly, genes of several other functional categories were also abundant in the Poribacteria SAG 3G transcriptome, including cell redox homeostasis related genes: superoxide dismutase (SOD2; 7.0 FPKM_HK_), thioredoxin (4.9 FPKM_HK_), and rubrerythrin (2.6 FPKM_HK_). Further, genes coding for membrane transport-associated proteins were highly expressed, particularly components of several ABC-transporters, biopolymer transporters, and the Sec dependent pathway translocation system. Notably, 11 transposase genes were present among the most expressed and 3 among the top 100. Few genes encoding Eukaryote-like repeat proteins (ELP), Bacterial Ig-like domains (n = 3) and Tetratricopeptide repeats (n = 2) were also highly transcribed, including TonB (1.7 FPKM_HK_) and the hypothetical protein CDS #2265144549 (1.4 FPKM_HK_). Additionally, the secondary metabolism related gene phosphotransferase ispE (2.6 FPKM_HK_), which encodes a protein that is part of the almost complete alternative nonmevalonate pathway for terpenoid biosynthesis, was highly transcribed. Finally, genes encoding phyH−domain containing proteins were abundant in the transcriptome, in particular with putative involvement in the biosynthesis of mitomycin antibiotics/polyketide fumonisin (1.3 FPKM_HK_; 1.1 FPKM_HK_). The functional elucidation of highly transcribed but poorly understood genes is an important undertaking to increase our understanding of Poribacteria physiology.

## Discussion

The present study provided novel, transcriptome-derived insights into poribacterial cell compartmentation as well as other highly expressed functions related to core metabolism and nutrient utilisation. A newly established microscopy protocol allowed the taxon-specific identification and 3D visualisation of Poribacteria within the extracellular sponge matrix as well as the subcellular localisation of highly transcribed poribacterial proteins involved in cell compartmentation. We combined here, to our knowledge for the first time, FISH and IHC with SEM on ultrathin sections of sponge tissue. The preparation of the samples with HPF allowed us to achieve superior cellular structure preservation over chemical fixation^33^,^34^ (Supplementary Figure S4). To date, there are only two FISH-CLEM protocols published both employing chemical sample fixation^35^,^36^. The presented FISH-IHC-CLEM toolset presents a significant step forward as it integrates taxonomic, functional, and structural information.

We consistently observed the ovoid-shaped morphotype with two granules in correlation with poribacterial-specific FISH signals, which may represent carbon-rich polymers such as poly-β-hydroxybutyrate (PHB)^37^ or glycogen^38^. This hypothesis is supported by the observed electron permeability of the structures since neither uranyl acetate nor lead citrate stain polysaccharides or polyesters. Moreover, their bipolar localisation is in agreement with descriptions for PHB-granules in other bacteria^39^. The ring-shaped fluorescent signals for Poribacteria specific FISH-probes that were originally observed by Fieseler, *et al*.^40^ using conventional microscopy might be attributed to the granules described here that might have caused probe exclusion.

**Figure 4:**
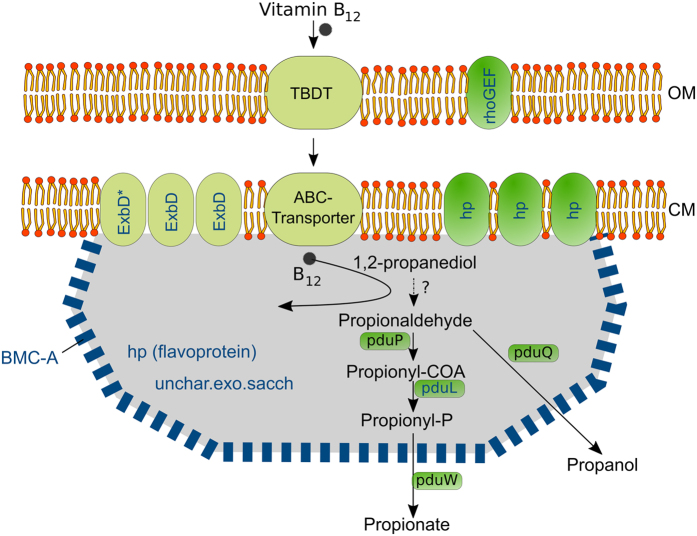
Hypothetic model for the BMC-A gene region function. Vitamin B-12 transport by Ton-B dependent systems occurs closely to reactions of the 1,2-propanediol degradation pathway, which is confined by BMC-shell like proteins. BMC-A encoded proteins are shown in blue color. Proteins are localised based on prediction (all but the BMC-shell marker), microscopic evidence (of BMC, and ExbD*) and literature (TonB-dependent transport system, Pdu proteins). OM, outer membrane; CM, cytoplasmic membrane; TBDT, TonB-dependent transporter; hp, hypothetical protein; unchar.exo.sacch, uncharacterized protein exopolysaccharide synthesis. Propenediol degradation pathway and localization of Pdu proteins relative to BMC-shell is assumed based on characterized BMCs^41^.

Bacterial microcompartments (BMCs) and their structural and functional diversity have received much recent attention^23^,^41^. Unlike the granules described above, they are protein-based and they contain enzymes and metabolic pathways. Here, we focused on the BMC-A genomic region that is structurally conserved among three poribacterial genomes (3G, 4CII and 4E) representing two distant clades^16^. These genomic regions are composed of CDSs encoding components of TonB-dependent periplasmatic energy transduction (ExbD, BPT), which are involved in biopolymer transport^42^, the outer membrane-predicted RhoGEF (COG5422), which is involved in the regulation of signal transduction pathways^43^, the propanediol utilisation protein PduL^44^, the BMC-shell marker protein (PF00936; 70% identity to PduA; SMTL id 4p2s.1), and several hypothetical proteins (Fig. 3). We showed that genes of the BMC-A region were highly transcribed with evidence of at least two polycistrons: one composed by BMC-shell marker and propanediol utilisation genes and the other composed by genes of the TonB-dependent energy transduction system. The functional relations within the BMC-A cluster genes were further supported by FISH-IHC-CLEM, where both the BMC-shell marker protein and ExbD* were co-localised at the cellular membrane (Fig. 3). In terms of functional relevance, we propose that the BMC-A region may be involved in 1,2-propanediol (1,2-PD) degradation (Fig. 4). The import of the cofactor vitamin B_12_^45^, may be driven by components of the TonB-system^42^, which are also encoded in the BMC-A gene region. Besides PduL, poribacterial SAGs encode further homologues of propanediol utilisation enzymes, which may convert propionaldehyde to propionate (exergonic reaction; PduP, PduW) or propanol (endergonic reaction, PduQ)^17^,^46^.

In conclusion, we obtained a better understanding of the candidate phylum Poribacteria biology by integrating information from different biological levels, i.e. DNA, RNA, protein, and cellular ultrastructure. Specifically, we identified poribacterial cells in the sponge tissue and studied their morphology, revealing the presence of characteristic bipolar granules possibly representing polymer depots. With respect to the function of the BMC-A region, the most conceivable hypothesis is that Poribacteria may perform propanediol utilisation reactions at the cytoplasmic membrane in areas confined by BMC-like proteins, including transformations of the toxic and volatile intermediate propionaldehyde. With regard to the methodological advances, the assembled microscopical FISH-IHC-CLEM toolset enables the simultaneous identification of specific microbes at high resolution in their environmental context, the study of their cellular structures, and the localisation of target proteins. Altogether, these methods contribute to and will facilitate an improved understanding of the uncultured environmental microorganisms.

## Methods

### Sample collection

*Aplysina aerophoba* individuals were collected by SCUBA diving in the Gulf of Piran (GPS: 45°31′N, 13°34′E), Piran, southwestern Slovenia, on May 15^th^, 2014 at 2 to 5 meters depth. Existing metatranscriptomes of the sponge *Xestospongia testudinaria* (Moitinho-Silva PhD thesis) were used for poribacterial transcriptome retrieval because all major poribacterial lineages were present in this dataset.

### Prokaryotic mRNA enrichment, sequencing and read processing

Prokaryotic mRNA was enriched from sponge total RNA and linearly amplified as previously described^32^. Sequencing was performed with Illumina HiSeq 2000 standard protocols, resulting in paired-end reads (101 bp) with an estimated mean insert size of 149 bp. The raw Illumina reads were processed according to Moitinho-Silva, *et al*.^32^. Briefly, (a) reads containing low quality bases were truncated to the first base below Phred score < 20; (b) sequencing adapters, including partial adapters, were trimmed; (c) remaining read pairs containing reads shorter than 16 bps were removed. Raw Illumina reads have been submitted to the National Center for Biotechnology Information under Biosample IDs SAMN02903553 (*XT*1), SAMN02903554 (*XT*2), and SAMN02903555 (*XT*3).

### Poribacteria transcriptome retrieval and gene expression estimation from metatranscriptomes

All six available single amplified genomes (SAGs) of Poribacteria (3G, 4C, 4CII, 4G, 4E and A3)^17^,^18^ were included in this study as reference for initial transcriptome abundance estimation. Genome-related files containing annotated sequence information were obtained from the Department of Energy (DOE) Joint Genome Institute (http://genome.jgi-psf.org). The success of the transcriptome retrieval procedure, i.e. mapping of metatranscriptomic reads to single-cell genomes, was optimised and validated based on a simulation experiment (see Suplementary Information, Section 1). Read mapping was performed with Bowtie2 v2.1.0 (Langmead & Salzberg, 2012), with the parameters: “--very-sensitive -I 20 -X 450”. In this step, unassembled, quality-processed, metatranscriptomic reads from 3 biological replicates were mapped to each poribacterial SAG. Read-mapping results in SAM format (Sequence Alignment/Map) were manipulated using SAMtools v0.1.18^47^.

To estimate transcript abundance, the reads aligned to coding sequences (CDS) were quantified using the htseq-count function of the HTSeq package^48^ in “no strand-specific” (-s no) and “union mode” (-m union). Non-uniquely mapped reads were discarded. The HTSeq “-samout” option was used to create SAM files, in which read pairs were uniquely assigned to a given CDS. Gene expression was estimated by normalising read counts to FPKM (Fragments Per Kilobase of exon, per Million fragments mapped), the paired-end equivalent of RPKM (Reads Per Kilobase of exon, per Million reads mapped), a measure used earlier^49^. In order to compare gene expression derived from the mapping of the three metatranscriptomic datasets, i.e. biological replicates, Pearson’s product-moment correlation, and standard deviation were calculated based on FPKM values in R v3.1.1^50^.

Among the poribacterial genotypes, 3G represented by far the most comprehensive transcriptomes retrieving 78.3% (2,345,508 of 2,995,024) of all sequences that were assigned to Poribacteria genomes (Supplementary Table S2). Besides, a proportion of 98.8% of SAG 3G genes was represented by the metatranscriptomic data set by at least one read-pair. Altogether, these results indicate a sufficient dynamic range for Poribacteria 3G transcriptional expression estimations^51^. Therefore, functional analyses of this study were based on Poribacteria SAG 3G. The gene functional annotations were based on the KEGG Ontology (KO)^52^, COG (clusters of orthologous groups, http://www.ncbi.nlm.nih.gov/COG/), and Pfam^53^ databases integrated with annotations deposited by Kamke, *et al*.^17^ at the Joint Genome Institute. Genomic regions were visualised using the Integrative Genomics Viewer^54^ and edited using Inkscape (https://www.inkscape.org).

## Microscopy

### HPF and freeze substitution

For high pressure freezing (HPF), *A. aerophoba* chimneys were dissected within 1 minute and placed into the 200 μm deep well of the freezing chamber (Specimen Carriers Type A (200 μm) and B (0 μm), Bal-Tec AG, Liechtenstein) filled with 1-hexadecene. Mesohyl samples were loaded into the HPF machine (EM HPM100, Leica Microsystems GmbH, Wetzlar, Germany) and cryo-immobilised at >20,000 K/s freezing speed and >2,100 bar pressure. Three specimens were processed for two sponge individuals. The freeze substitution protocol, as adapted from Weimer^55^, the embedding procedure, and the sectioning protocol are provided in the Supplementary Information, Sections 2 and 3.

### Fluorescence *in situ* hybridisation (FISH) for FISH-CLEM

Poribacterial cells were identified within the *A. aerophoba* tissue by *in situ* hybridisation on ultrathin LR-white embedded-array-sections. Poribacteria 16S rRNA was hybridized using the double labelled probe POR1130 (5′-[Alexa546]GGC TCG TCA CCA GCG GTC[Alexa546]-3′; Fieseler, *et al*.^40^) at a concentration of 7 ng/μl. Hybridisation took place within Sylgard chambers (in-house production) inside an equilibrated humid chamber at 46 °C for 3 h in hybridisation buffer (900 mM NaCl, 20 mM Tris/HCL pH 7.4, 30% formamide, 0.01% sodium dodecyl sulphate). For counter-staining of bacterial nucleic acids and sponge cell nuclei, the hybridisation solution was exchanged with pre-warmed DAPI in hybridisation buffer (1 ng/μl), followed by 20 min incubation at 46 °C. After this, the arrays were incubated in pre-warmed wash buffer (20 mM Tris/HCL; 112 mM NaCl, 5 mM EDTA; 0.005% sodium dodecyl sulphate) at 48 °C for 25 min. Finally, the slides were carefully rinsed with a laminar flow of ice cold ddH_2_O and were directly mounted in Mowiol medium (Mowiol^®^ 40–88, Kuraray Europe GmbH, Tokyo, Japan). In addition to Poribacteria-specific probes, FISH was performed with a Chloroflexi probe (sponge cluster I, GNS934, Alexa488, 10 ng/μl). No co-localisation was observed indicating specificity of the Poribacteria probe. The POR1130 sense probe (5′-[Alexa546]GAC CGC TGG TGA CGA GCC[Alexa546]-3′) was used as a control for false positive staining and did not show detectable signals.

### Antibody design and immunohistochemistry (IHC)

Affinity purified polyclonal antibodies (Genscript, NJ, USA) were raised in rabbit based on peptides of highly transcribed poribacterial SAG 3G genes. Peptides were selected aiming for maximum antigenicity (OptimumAntigen™ Design Tool; Genscript) and minimum host similarity. Additionally, peptides with less than 60% identity to other poribacterial proteins were chosen. For each target protein, two (BMC-shell marker protein, ID 2265142951) or three (ExbD protein, ID 2265142941; gas vesicle protein, ID 2265144305) peptides were picked for antibody production. The immunological staining procedure was adapted from Micheva and Smith^28^ with modifications (Suplementary Information, Section 4). Arrays only incubated with secondary antibody were used as a negative control, showing few background fluorescence signals. The sensitivity of the primary antibodies was confirmed by immuno-dot-blotting. Monoclonal b-tubulin (mouse) antibody was used as positive control during protocol standardisation.

### Scanning electron microscopy preparations

After the light microscopic images were taken, the cover slip was carefully removed with a razor blade and the whole slide was washed in ddH_2_O to remove the mounting medium. After drying, the sections were contrasted in 2.5% uranyl acetate in ethanol for 15 min and in 50% Reynolds’ lead citrate^56^ in boiled ddH_2_O for 10 min. The slides were size-reduced with a diamond pen and attached to a scanning electron microscopy (SEM) pin stub specimen mount. Electrically conductive adhesive was added to one side of the glass piece to allow electron flow from the surface to the specimen mount. Finally, the sample was coated with a carbon layer to prevent charging of the sample.

### Image acquisition

The fluorescence signals of IHC and FISH were captured using the ELYRA S.1 superresolution structured illumination microscope (Zeiss, Göttingen, Germany) and the Axio Observer.Z1 microscope (Zeiss, Göttingen, Germany), respectively. In order to follow regions of interest on consecutive sections by fluorescence- and electron microscopy, reference maps were established based on relative positions to section edges and structures with large and consistent z dimension. Initial processing of the obtained fluorescence images was carried out with the ImageJ distribution Fiji^57^,^58^. Briefly, background signal levels were determined as average maximum intensities of three cell-free mesohyl regions, the brightness and contrast were adjusted accordingly and custom lookup tables were applied. On the same sections, that were used for fluorescence microscopy, SEM was carried out using a field emission scanning electron microscope JSM-7500F (JEOL, Japan) with LABE detector (for back scattered electron imaging at extremely low acceleration voltages) directly on the microscope slides.

### Correlation and set alignment

Using the rough reference map described above, regions of fluorescence microscopy were identified at SEM resolution based on sponge heterochromatin patterns. The obtained z-stacks of fluorescence microscopy and SEM images were automatically aligned in TrackEM2^59^ using the align layers function in least square mode with 8 steps per octave, a maximum image size of 3,000 pixels and rigid mode for feature extraction whilst allowing a maximum alignment error of 100 pixels. The applied desired transformation was rigid and affine for light and electron micrographs, respectively. For FISH-CLEM, the aligned stacks were collectively correlated based on the middle serial section of an array. In order to enable the correlation precision required for high resolution IHC fluorescence images, IHC-CLEM correlation was established using the Fiji implemented Landmark Correspondences plugin (moving least squares; mesh resolution 200; affine), referencing characteristic features of both fluorescence and electron microscopy, such as sponge nuclei heterochromatin. The correlation of FISH and IHC with electron microscopy is termed “FISH-IHC-correlative light and electron microscopy” (FISH-IHC-CLEM). For the combination of IHC and FISH, in the current setup, images were taken on consecutive sections of 100 nm distance with alternating protocols (3 slices IHC; 1 slice FISH; 3 slices IHC pattern). The segmentation, 3D tomographic reconstruction and subsequent analysis of consecutive FISH-CLEM sections was carried out using the IMOD software package v.4.7^60^.

## Additional Information

**How to cite this article**: Jahn, M. T. *et al*. Shedding light on cell compartmentation in the candidate phylum Poribacteria by high resolution visualisation and transcriptional profiling. *Sci. Rep.* **6**, 35860; doi: 10.1038/srep35860 (2016).

**Publisher’s note:** Springer Nature remains neutral with regard to jurisdictional claims in published maps and institutional affiliations.

## Supplementary Material

Supplementary Dataset

Supplementary Information
