# Supplementary material for: Shedding light on cell compartmentation in the candidate phylum Poribacteria by high resolution visualisation and transcriptional profiling
Source: Sci Rep. 2016 Oct 31;6:35860. doi: 10.1038/srep35860 (PMC5087111; doi:10.1038/srep35860)
Supplement: Supplementary Information [file srep35860-s2.pdf]

Shedding light on cell compartmentation in the candidate phylum Poribacteria by high resolution visualisation and transcriptional profiling

Martin T Jahn[1,2], Sebastian M Markert[3], Taewoo Ryu[4], Timothy Ravasi[4], Christian Stigloher[3], Ute Hentschel[1,2], and Lucas Moitinho-Silva[2,5]

[1] Present address: Marine Microbiology, GEOMAR Helmholtz Centre for Ocean Research, Kiel, 24105, Germany

[2] Julius-von-Sachs Institute for Biological Sciences, University of Würzburg, Würzburg, 97082, Germany

[3] Division of Electron Microscopy, Biocenter, University of Würzburg, 97074, Würzburg, Germany.

[4] Division of Biological and Environmental Sciences & Engineering, King Abdullah University of Science and Technology, Thuwal, 23955-6900, Kingdom of Saudi Arabia

[5] School of Biotechnology and Biomolecular Sciences & Centre for Marine Bio-Innovation, University of New South Wales, Sydney, 2052, Australia

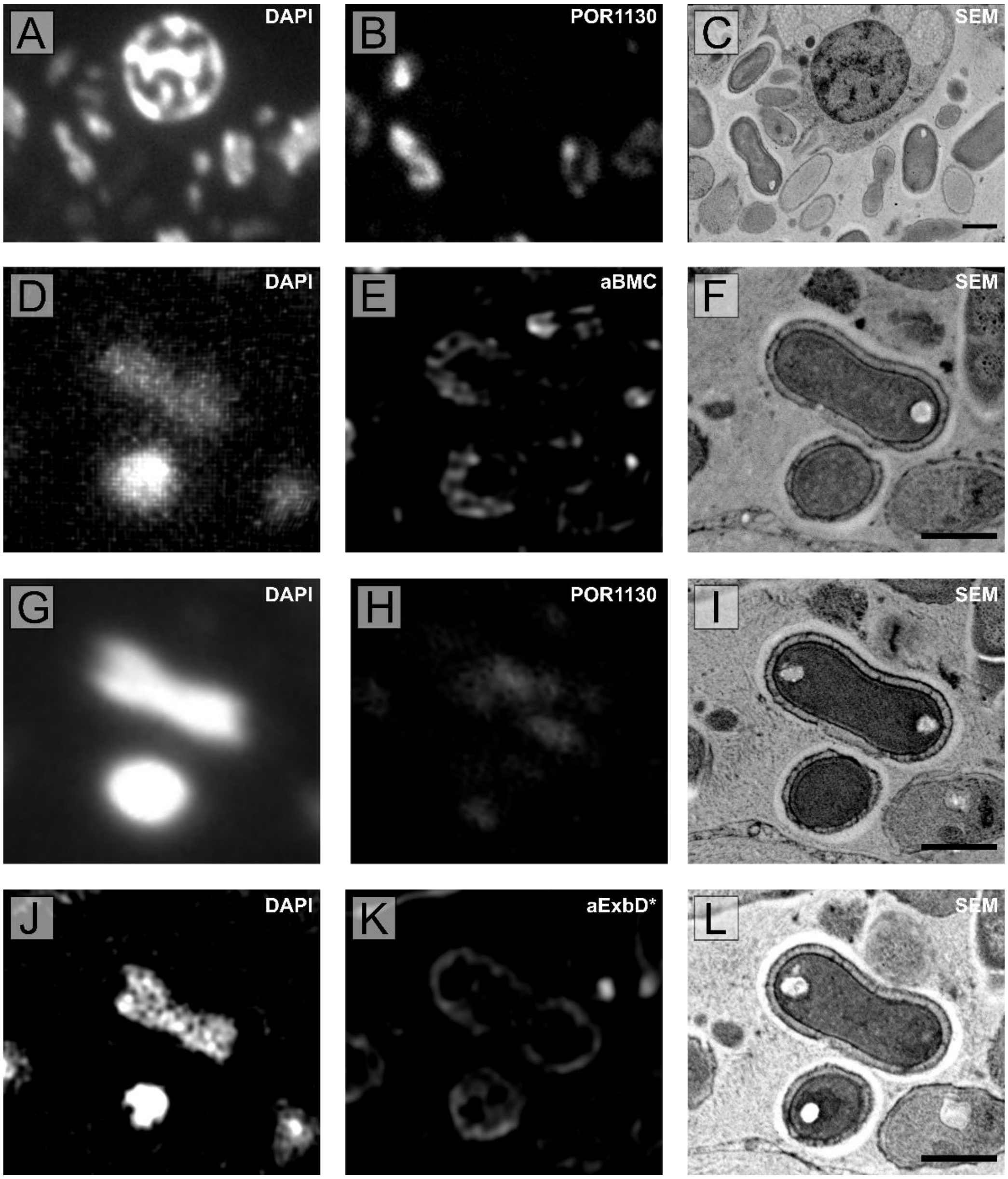

Supplementary Figure S1: Separate channels for Figure 1a (A-C) and Figure 3b (D-L) of the main text. (A) Fluorescence signal of the nucleotide stain DAPI, **(B)** the Poribacteria specific 16S rRNA probe POR1130 (Alexa546) and **(C)** scanning electron microscopy image (SEM) of a LR-white section. Scale bar, 1 µm. Panel rows **D-F**, **G-I, J-L** represent DAPI staining **(D, G, J)** and specific staining of **(E)** BMC-shell marker (FITC), **(H)** Poribacteria 16S rRNA (POR1130; Alexa546) and **(K)** ExbD protein (FITC) on three consecutive sections (100nm). **(F, I, L)** The corresponding SEM images of the same sections. Scale bar, 500 nm.

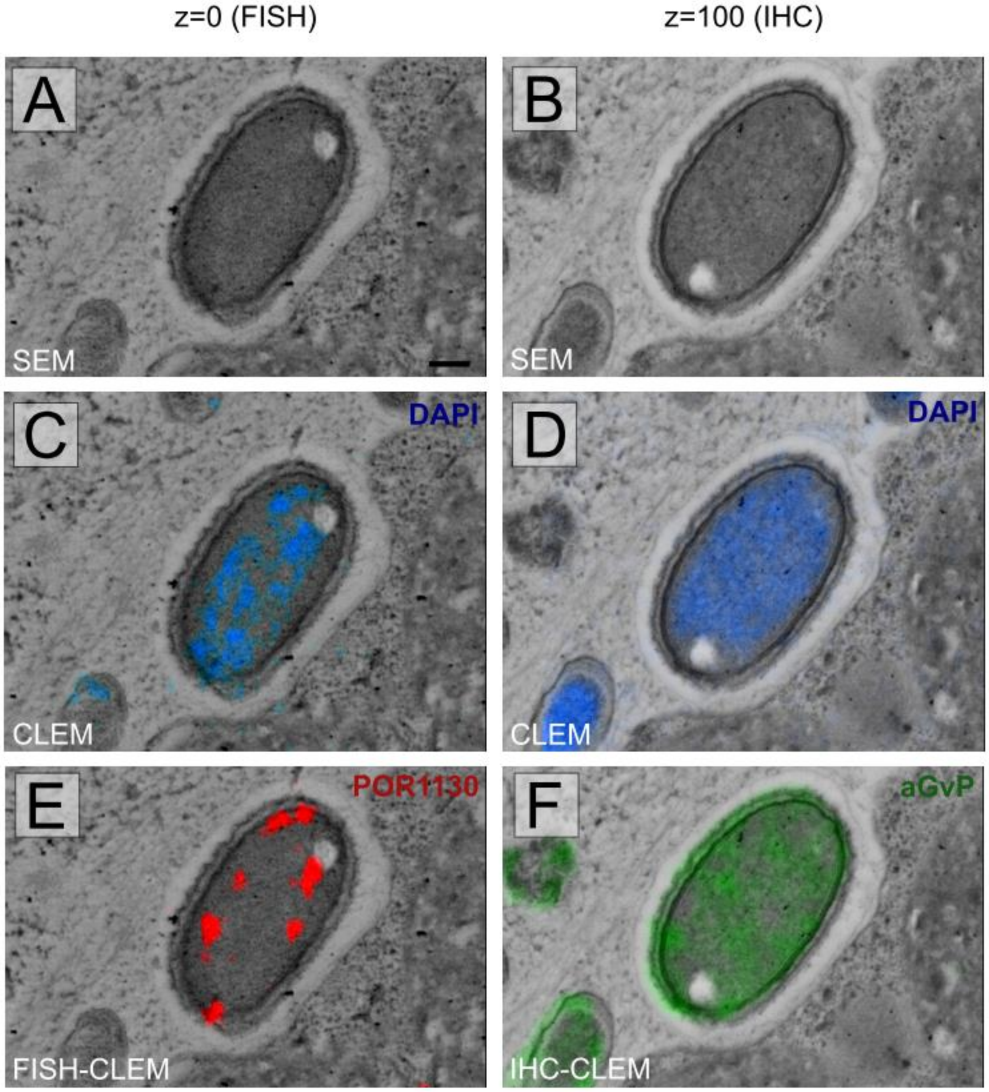

Supplementary Figure S2: FISH-IHC-CLEM identification of a poribacterial cell and localisation of poribacterial gas vesicle protein (GvP) on ultrathin *A. aerophoba* sections. **(A-B)** SEM images of two consecutive sections of 100 nm distance at 33,000 magnification. **(C-D)** DNA is counterstained with DAPI (blue). The consecutive sections were stained **(E)** with the double labelled probe POR1130 (red; Alexa546; 5'3'), specific for Poribacteria **(F)** and with FITC labelled donkey anti-rabbit antibody directed against three anti-GvP peptide antibodies (green). Scale bars, 100 nm.

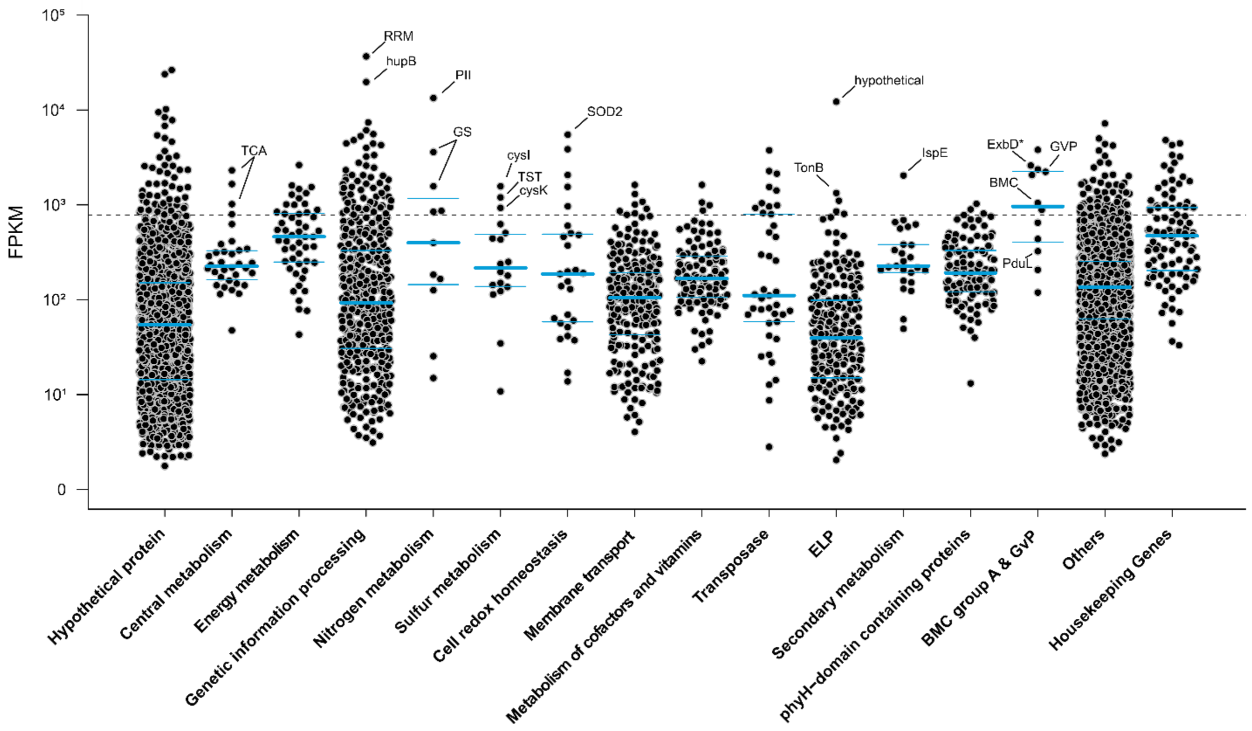

Supplementary Figure S3: Active functions of poribacterial SAG 3G in the sponge *X. testudinaria.* Expression estimations (FPKM) are shown for genes of selected functional categories. Blue horizontal lines indicate the first and third quartiles and the median (thick line) of each category. Dashed line indicates average expression level of housekeeping genes. Genes highlighted are citrate cycle genes (TCA), RNA recognition motif (RRM), DNA-binding protein HU (heat unstable)-beta (hupB), nitrogen regulatory protein PII, glutamine synthases (GS), thiosulfate sulfurtransferase (TST), NADPH-dependent sulfite reductase (cysI), cysteine synthase A (cysK), superoxide dismutase (SOD2), TonB protein, ispE phosphotransferase, ExbD*, gas vesicle protein (GvP), BMC-shell marker, and propanediol utilisation protein (PduL). The FPKM values represent the average of three biological replicates.

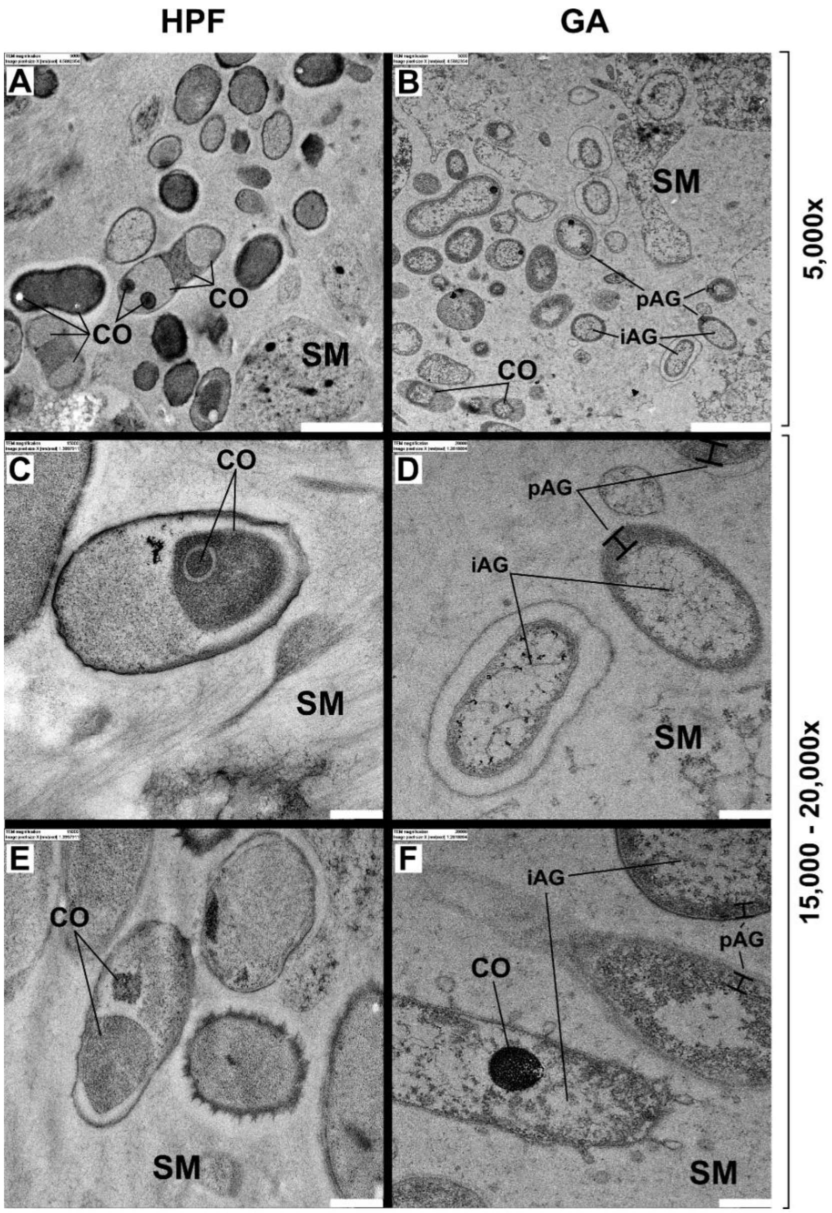

Supplementary Figure S4: Preservation of *A. aerophoba* mesohyl and associated microorganisms by high pressure freezing (HPF) compared to glutaraldehyde (GA) fixation. At different magnifications, representative HPF electron micrographs **(A,C,E)** obtained by a JEOL JEM-2100 transmission electron microscope (Jeol, Japan) were compared with those obtained after GA fixation **(B,D,F)** performed according to Fieseler, et al. [1]. SM, sponge mesohyl; CO, compartment like structures; iAG, internal electron dense filiform agglomerations; pAG; peripheral electron dense agglomerations. Scale bar A, B 2 µm; C, D, E, F 400 nm.

	Biological replicate		
	*XT*1	*XT*2	*XT*3
Sequenced raw reads (paired-end)	44,692,742	63,488,358	56,290,470
Ave. raw read length (bp)	101	101	101
Estimated insert size (bp)	150	148	148
Quality filtered reads (paired-end)	35,049,638	50,716,436	43,464,006
Quality filtered bases (bp)	2,644,836,363	3,818,929,178	3,296,358,374
Ave. quality filtered read length (bp)	75 ± 22.46	75 ± 22.26	75 ± 22.05
GC- content of sequenced library (%)	53.1	53.5	53.7

Abbreviations: XT, *Xestospongia testudinaria*; Ave., average; ±, strandard deviation

Table S1: Summary statistics for *X. testudinaria* metatranscriptome datasets

Poribacterial clade	Genome size (bp)	Biological replicate	Mapped reads (% of meta-transcriptome)	Mapped reads inside CDS (% of aligned transcriptome)	Mapped reads inside non-coding DNA (rDNA, tDNA) (% of aligned transcriptome)	Average CDS coverage ± sd
3G	5,441,554	XT1	1,417,774 / 4.05%	866,433 / 61.1%	551,341 / 38.9%	14.4 ± 56.3
		XT2	1,582,793 / 3.12%	737,915 / 46.6%	844,878 / 53.4%	11.9 ± 77.7
		XT3	1,584,119 / 3.64%	741,160 / 46.8%	842,959 / 53.2%	12.1 ± 57.6
4C	1,629,923	XT1	309,285 / 0.88%	10,192 / 3.3%	299,093 / 96.7%	0.4 ± 3.9
		XT2	421,848 / 0.83%	15,534 / 3.7%	406,314 / 96.3%	0.6 ± 4.3
		XT3	677,071 / 1.56%	10,549 / 1.6%	666,522 / 98.4%	0.4 ± 2.6
4CII	543,453	XT1	406,377 / 1.16%	79,460 / 19.6%	326,917 / 80.4%	12.7 ± 42.8
		XT2	500,483 / 0.99%	62,688 / 12.5%	437,795 / 87.5%	9.7 ± 44.6
		XT3	774,492 / 1.78%	64,815 / 8.4%	709,677 / 91.6%	10.2 ± 35.2
4G	189,191	XT1	51,615 / 0.15%	28,871 / 55.9%	22,744 / 44.1%	12.9 ± 51.5
		XT2	80,857 / 0.16%	52,110 / 64.4%	28,747 / 35.6%	23.2 ± 92.7
		XT3	71,426 / 0.16%	38,485 / 53.9%	32,941 / 46.1%	16.7 ± 87.4
A3	414,219	XT1	41,177 / 0.12%	24,317 / 59.1%	16,860 / 40.9%	4.8 ± 45.3
		XT2	60,684 / 0.12%	43,483 / 71.7%	17,201 / 28.3%	8.9 ± 95.9
		XT3	54,420 / 0.13%	29,737 / 54.6%	24,683 / 45.4%	6.0 ± 56.7
4E	3,647,669	XT1	207,155 / 0.59%	49,347 / 23.8%	157,808 / 76.2%	0.9 ± 3.7
		XT2	311,112 / 0.61%	87,841 / 28.2%	223,271 / 71.8%	1.7 ± 7.0
		XT3	283,139 / 0.65%	52,087 / 18.4%	231,052 / 81.6%	0.9 ± 3.7

Abbreviations: ±, standard deviation

Table S2: RNA-Seq mapping summary

1. Optimisation and validation of in silico transcriptome retrieval

To evaluate the specificity and sensitivity of the transcriptome retrieval strategy from bacterial community metatranscriptomes we used simulated reads for which we knew the correct alignment. Using Mason v0.1.2 [2] we simulated sets of 20-30 Mio Illumina-like paired-end reads 75 nt long. In an optimisation phase, transcriptomic reads were simulated from poribacterial sequences as target and the Sargasso Sea metagenome (AACY000000000.2; Venter et al 2004) as transcriptional background. The ratio target:background used was 1:10, which was assumed based on Poribacteria relative abundance in amplicon sequencing data [3]. On this read dataset we ran (i) a commercial: Geneious v6.0.6 (Biomatters Ltd., Auckland, New Zealand); (ii) a "spaced" seed-based: Shrimp2 v2.2.3 [4]; and (iii) a Burrows-Wheeler-Transformation (BWT): Bowtie2

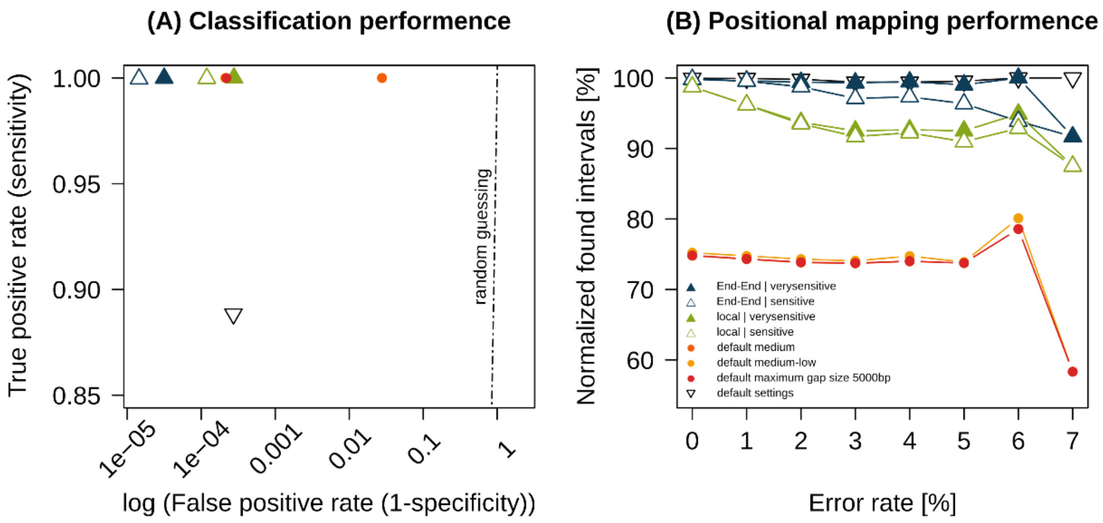

Figure 1: Evaluation of mapping software and parameters with simulated test data. (A) Two dimensional ROC space with eight discrete classifiers illustrating the relative trade- off between sensitivity and specificity of read mapping (levels: mapped/not mapped). The dashed diagonal line represents performance of random training free guess. (B) Normalised found intervals of software-parameters of simulated reads. This metric reflects the proportion of reads that were accurately mapped to the correct intervals, while down-weighting mapping to multiple positions. ▲, • and ▽ represent the mapping software Bowtie2, Geneious and Shrimp2, respectively. The legend key is the same for all plots.

v2.1.0 [5] read aligner using different settings (**Figure 1**). The NFI-metrics were computed with Rabema v1.2.0 [6]. Bowtie2 in very-sensitive mode (-I 20 -X 450) was the setup that combined high alignment specificity and sensitivity with error tolerant positional mapping entered the validation phase of the simulation.

Here, taxon level dependent performance of the transcriptome retrieval strategy was accessed to investigate the effect of the taxonomic distance between the target and the metatranscriptomic backround. For this task, 27 transcriptomes were drawn from random bacterial genomes and tested each against a simulated metatranscriptomic background drawn from 399 randomly

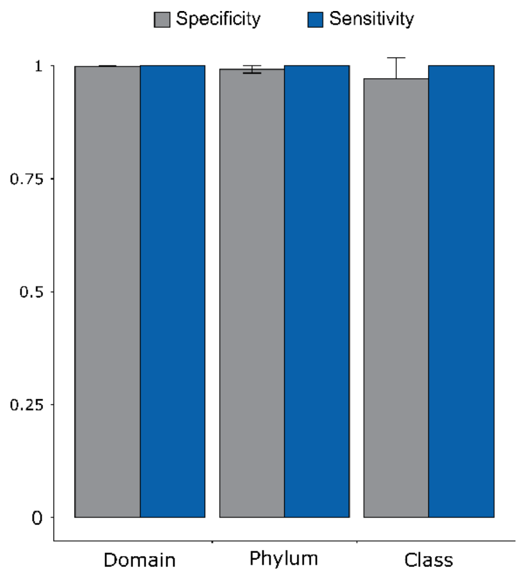

Figure 2: Specificity and sensitivity of transcriptome retrieval for different taxonomic ranges of simulated communities. For each taxonomic rank simulations were iterated 9 times. The used confusion matrix was: target read-mapped=true positive; target read-unmapped= false negative; background read-mapped= false positive; background read-unmapped= true negative. Error bars indicate s.d.

selected genomes of the same taxon rank as the respective target, i.e. domain, phylum or class (obtained from Genomes OnLine Database GOLD, http://www. genomesonline.org/). The success of transcriptome retrieval for the taxonomic ranges is given in **Figure 2**. Optimised read-

mapping parameters thus allow for high sensitivity and specificity for investigations of transcriptional expression at the phylum level.

2. Freeze-substitution and embedding protocol for FISH-IHC-CLEM

The freeze substitution protocol was adapted from Weimer [7]. Specifically, high pressure frozen samples in liquid nitrogen were directly transferred to an EM AFS2 freeze substitution system (Leica Microsystems, Wetzlar, Germany) and incubated in 0.1% $KMnO_4$ in anhydrous acetone at -90°C for 80 h. After the first hour, the solution was exchanged once. Next, the temperature was ramped for 11 h to -45°C and the samples were thoroughly washed 4-5 x with anhydrous acetone over the course of 3 h. Acetone was exchanged with ethanol through an ethanol series (30 min 32% ethanol in acetone, 30 min 64% ethanol in acetone, 2 x 30 min 96% ethanol in water). Then the temperature was ramped for 16 h to 4°C and the samples were washed again 2 x with 96% ethanol. For embedding, the solution was substituted with LR White (Medium Grade Acrylic Resin, London Resin Company Ltd.) in several steps as follows: First, the samples were incubated overnight at 4°C in 50% LR White in ethanol. Subsequently, samples were washed 3 x in 100% LR White (with incubation times of 1 h, 4 h, and overnight) at 4°C. Finally, specimens were transferred to RT and washed after 3 h directly before embedding in LR White resin and subsequent curing for 48 h at 52°C.

3. Sectioning protocol

Thin sections of 100 nm were cut with a Leica EM UC7 ultramicrotome (Leica Microsystems, Wetzlar, Germany) using a Histo Jumbo Diamond Knife (Diatome AG, Biel, Switzerland). A microscope slide coated with poly-L-lysine (Polysine slides, Thermo Fisher Scientific, Waltham, MA, USA) was submerged in the knive's water tray before cutting to receive the sections. To obtain ribbons, a thin layer of a 1:1 mixture of glue (Pattex Kraftkleber

Gel/Compact, Henkel, Düsseldorf, Germany) and xylene was applied to the lower side of the block. Slides with sections were dried at 50°C for at least 20 min before staining. Slides not used immediately were stored in a dust-free and dry environment at room temperature.

4. Immunological staining procedure for FISH-IHC-CLEM

The staining procedure was adapted from Micheva and Smith [8] with modifications. PAP pen (Liquid Blocker, Japan) encircled arrays on microscope slides were placed into a humid chamber and incubated for 10 min with 50mM glycine in Tris-buffer (0.05 M Tris, 0.15 M NaCl, pH 7.6). Except when otherwise mentioned, all incubations were carried out at room temperature. The arrays were blocked using BSA blocking solution (0.1% BSA, 0.05% Tween in Tris-buffer) for 20 min. During blocking time, peptide antibodies for one target protein were diluted and pooled (2x aBMC; 3x aExbD, 3x aGvP) in blocking solution to 8 µg/ml with subsequent centrifugation at 13,000 rpm (Mikro 20 centrifuge; Hettich, Germany) for 2 min. The arrays were incubated with primary antibodies for 2 h. The arrays were rinsed for 15 sec and washed during 5 min with Tris-buffer for four times. Prior to application, the secondary tetramethylrhodamine isothiocyanate (TRITC) fluorophore labelled donkey anti-rabbit antibody (Jackson ImmunoResearch Laboratories, USA) was diluted 1:100 in blocking solution and incubated with *A. aerophoba* tissue overnight at 4°C. The secondary antibody was recovered from the supernatant after centrifugation for 3 min and 13,000 rpm. This procedure was carried out to reduce false positive binding of the secondary antibody. Each array was incubated with the subtracted secondary antibody for 30 min in the dark. The arrays were washed as described above and incubated with 1 ng/µl DAPI in Tris-buffer for 20 min. Finally, the arrays were washed and rinsed with ddH_2O before mounting in Mowiol.

REFERENCES

1 Fieseler, L., Horn, M., Wagner, M. & Hentschel, U. Discovery of the novel candidate phylum "Poribacteria" in marine sponges. *Appl Environ Microbiol* **70**, 3724-3732 (2004).

2 Holtgrewe, M. Mason–a read simulator for second generation sequencing data. *Technical Report FU Berlin* **962** (2010).

3 Moitinho-Silva, L. *et al.* Specificity and transcriptional activity of microbiota associated with low and high microbial abundance sponges from the Red Sea. *Mol Ecol* **23**, 1348-1363 (2014).

4 David, M., Dzamba, M., Lister, D., Ilie, L. & Brudno, M. SHRiMP2: sensitive yet practical SHort Read Mapping. *Bioinformatics* **27**, 1011-1012 (2011).

5 Langmead, B. & Salzberg, S. L. Fast gapped-read alignment with Bowtie 2. *Nat Methods* **9**, 357-359 (2012).

6 Holtgrewe, M., Emde, A. K., Weese, D. & Reinert, K. A novel and well-defined benchmarking method for second generation read mapping. *BMC Bioinformatics* **12**, 210 (2011).

7 Weimer, R. M. Preservation of C. elegans tissue via high-pressure freezing and freeze-substitution for ultrastructural analysis and immunocytochemistry. *Methods Mol Biol* **351**, 203-221 (2006).

8 Micheva, K. D. & Smith, S. J. Array tomography: a new tool for imaging the molecular architecture and ultrastructure of neural circuits. *Neuron* **55**, 25-36 (2007).
